# The loading patterns of a short femoral stem in total hip arthroplasty: gait analysis at increasing walking speeds and inclines

**DOI:** 10.1186/s10195-018-0504-0

**Published:** 2018-08-17

**Authors:** Anatole V. Wiik, Mads Brevadt, Hardeep Johal, Kartik Logishetty, Oliver Boughton, Adeel Aqil, Justin P. Cobb

**Affiliations:** Imperial College London, MSK Lab, Department of Surgery and Cancer, Charing Cross Hospital, Fulham Palace Road, London, W6 8RF UK

**Keywords:** Gait, Treadmill, Weight acceptance, Hip arthroplasty

## Abstract

**Background:**

The purpose of this study was to examine the gait pattern of total hip arthroplasty (THA) patients with a new short femoral stem at different speeds and inclinations.

**Materials and methods:**

A total of 40 unilateral THA patients were tested on an instrumented treadmill. They comprised two groups (shorter stemmed THA *n* = 20, longer stemmed THA *n* = 20), both which had the same surgical posterior approach. The shorter femoral stemmed patients were taken from an ongoing hip trial with minimum 12 months postop. The comparative longer THR group with similar disease and severity were taken from a gait database along with a demographically similar group of healthy controls (*n* = 35). All subjects were tested through their entire range of gait speeds and inclines with ground reaction forces collected. Body weight scaling was applied and a symmetry index to compare the implanted hip to the contralateral normal hip. An analysis of variance with significance set at *α* = 0.05 was used.

**Results:**

The experimental groups were matched demographically and implant groups for patient reported outcome measures and radiological disease. Both THA groups walked slower than controls, but symmetry at all intervals for all groups were not significantly different. Push-off loading was less favourable for both the shorter and longer stemmed THR groups (*p* < 0.05) depending on speed.

**Conclusions:**

Irrespective of femoral stem length, symmetry for ground reaction forces for both THA groups were returned to a normal range when compared to controls. However individual implant performance showed inferior (*p* < 0.05) push-off forces and normalised step length in both THR groups when compared to controls.

**Level of evidence:**

III.

## Introduction

The treatment of end-stage hip arthritis with arthroplasty in the young or active carries a concerning burden of revision due to improved life expectancy [1]. Total hip arthroplasty (THA) utilising a shorter femoral stem has been proposed to be useful in tackling this challenge. Shorter femoral stems have been shown to better load the proximal metaphyseal bone, improving proximal implant fixation and osseointegration as well as reducing bone loss from stress shielding [2, 3]. Proponents of their use also declare a lower incidence of mid-thigh pain, as by proximity, the tip of the short stem is less likely to abut the diaphyseal endo-cortices [4, 5]. The more physiological loading resulting from short stem use may be the reason why there is an emergence of studies demonstrating they may be protective against the risk of a periprosthetic fracture when compared with longer uncemented stem designs [6].

Short stem hip implants have been around since 1938 developed by Wiles at the Middlesex hospital [7] but its worldwide use has been credited to Pipino [8]. The development of recent classification systems for stems based on their length, emphasises the great diversity of hip stems lengths which are now available [9, 10]. Current mid-term results are comparable to those of successful long stems designs and with reported survival rates greater than 95% [11, 12]. Reassuringly, radiographical, subsidence seen with some short stem designs, are also similar to those of long stems [13].

Despite the obvious advantages conferred by a short stem, the question as to what function patients should expect to achieve following the implantation of these devices remains relatively unaddressed. A recent prospective study utilising patients reported outcome measures (PROMS), attempted to shed light on this subject, however the ceiling effect limited its “no difference” conclusion [14]. Gait analysis, on the contrary, does not suffer a ceiling effect and would allow one to observe hip function in everyday life [15]. Furthermore gait assessment has demonstrated important loading changes that occur after hip arthroplasty [16].

To objectively determine the loading behaviour of a short femoral stem, a study examining the entire range of gait speed and incline was sought for a short stem THA. The aim of this gait study was threefold. First we wished to determine the impact femoral stem length has on symmetry with ground reaction forces, by comparing it the contralateral “normal” side. Secondly we wanted to compare the performance of a geometrically similar shorter stemmed device with an established ODEP (orthopaedic device evaluation panel) rated 10A* longer stemmed implant. Lastly, and in order to put findings into context we also aimed to compare the gait of the operated limb with those of an asymptomatic and matched control group. We hypothesised that a short femoral stem device would allow a near-physiological gait at higher speeds by decreasing the stiffness of the femoral shaft.

## Materials and methods

**Fig. 1:**
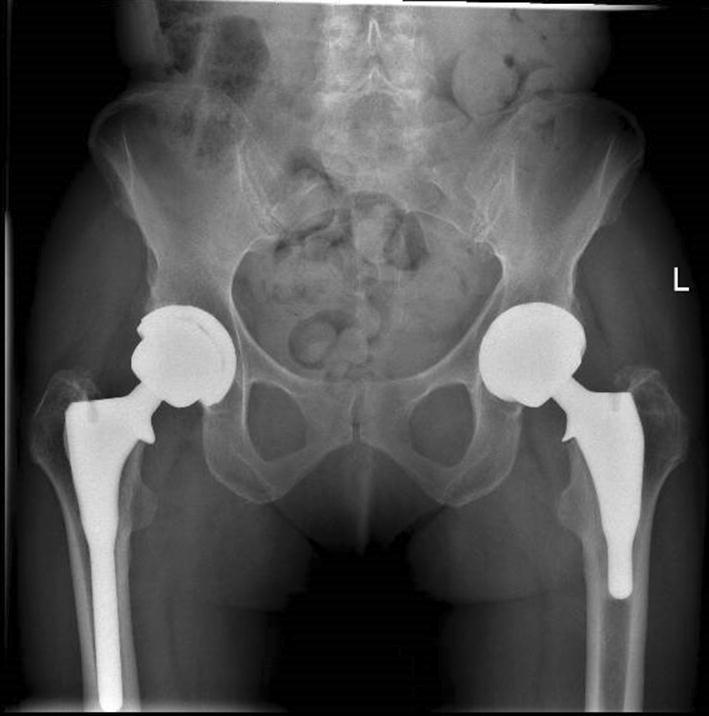
Implant design comparison

All short stem THA subjects were identified from the multicentre and ongoing Evolution Hip Trial [17] database site with gait analysis. Following study ethical approval, consenting subjects had their gait assessed using a treadmill instrumented with force plates. A total of 20 patients from the trial database were identified as meeting the inclusion criteria of having an ipsilateral uncemented short stem hip replacement in situ, and of being a minimum of 12 months following surgery and having a full gait data set walking on the flat and uphill to evaluate. To compare, we identified all patients (*n* = 20) on the same gait database having the same inclusion/exclusion criteria having had the predecessor uncemented longer stem. We obtained an age and sex matched group of asymptomatic normal controls (*n* = 35) from our previously tested database, so this meant that 75 subjects were analysed. All hip arthroplasty subjects were implanted with a geometrically similar hydroxyapatite stem which differed only for length (Fig. 1), (Furlong HAC and Furlong Evolution, Joint Replacement Instruments, Sheffield, England). Both stems are ODEP approved, with the Furlong HAC, which has been around longer, having the best possible, 10A*, rating [18]. Case records of the arthroplasty group were further screened to confirm an uncomplicated surgical recovery and ensure the absence of any other lower limb replacements or conditions, which might have affected walking ability. All subjects were analysed by a blinded assessor to avoid any potential testing bias.

### Radiological assessment

Radiographic pre-operative disease severity was assessed using Ahlback’s grading system and orthogonal radiographs of the hip [19]. Postoperative radiographs were scrutinised to ensure that an accurate reconstruction of hip off-set, leg length and cup inclination had been achieved [20].

### Surgical intervention and rehabilitation

All surgery was performed by the senior surgical author (JPC) using the same posterior approach with a trans-osseous muscle and capsular repair for all THA. The senior surgeons’ implant of choice switched from the long to the short stem following its introduction. Thus the long stem patients were operated earlier than the currently trialled short stem device and had a longer follow-up. All THA patients had undergone a standard rehabilitation programme irrespective of the implant used.

### Patient reported outcome measures (PROMS)

PROMS assessment in the form of the Oxford hip (OHS) [21], EuroQol 5 part questionnaire (EQ-5D) with the EuroQol visual analogue scale (EQ-VAS) scores [22] were obtained post-operatively. Subject demographics including height and weight were also recorded.

### Symmetry index

The validated symmetry index assessed the gait symmetry of the implanted limb to the contra-lateral normal limb [23]. It was calculated using the formula:

$${\text{Absolute SI}}\, = \,\frac{{\left| {X1\, - \,X2} \right|}}{{0.5 \, \times \,\left( {X1\, + \,X2} \right) }} \, \times \,100\%$$

where *X*1 was the implanted limb measure and *X*2 was the contra-lateral normal limb measure [24]. It gave a measure of percent difference between limbs. *X*1 and *X*2 was used for controls right and left respectively.

### Gait analysis

**Fig. 2:**
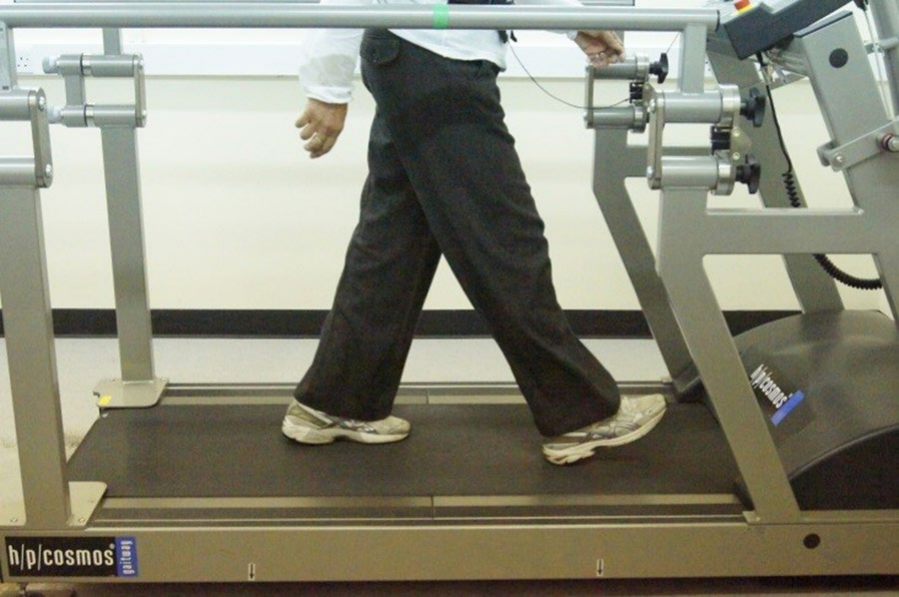
Weight acceptance phase on the instrumented treadmill

Gait performance was tested using a treadmill (Fig. 2) instrumented (Gaitway™ II, Kistler Instrument 104 Corporation, Amherst NY) using a protocol previously described [16]. Hof scaling and body weight normalising was performed to correct for subject height and mass differences [25]. As a 10 s sampling interval collected data for a number of steps for each limb, outputted GRFs were subject to averaging using a custom written MATLAB script. Data was stratified into short and long stems for the replacement groups and right and left limbs for the healthy control group. GRF variables analysed included: weight acceptance, mid-stance, and push-off. Step length was also analysed with it being normalised for height.

### Statistical analysis

Statistical analysis was performed with Matlab (Matworks). All variables were shown to be normally distributed with a Shapiro–Wilk test. All variables for each of the subject group were compared to each other using an analysis of variance (ANOVA) with Tukey post hoc test with significance set at *α* = 0.05. Also, for continuous variables between implanted limbs, an independent *t* test was used with significance set at *α* = 0.05. A Chi-squared test was used for categorical variables (gender and pre-operative x-ray diagnosis).

## Results

The arthroplasty and control groups were matched for gender, BMI and height (Table 1). PROMs revealed excellent post-operative patient scores and satisfaction as measured using the OHS and Eq-5D. As anticipated, the short stemmed hip replacements had a significantly (*p* < 0.001) shorter follow-up period (13 vs 21 months) as it is under trial currently. Hips of all patients had normal pelvic morphology and had simple primary OA. Both sides had similar preoperative disease severity. In all arthroplasty subjects the implant positions in terms of hip offset and leg length were within 5 mm of each other.

**Table 1:** Subject characteristics, radiographic OA severity, patient reported outcome measures

Subject	Short stem	Long stem	Control
Sex M:F	5:15	8:12	15:20
Age (yrs)	69 (38–84)^†‡^	63 (31–86)	61 (41–85)
BMI	26 (17–35)	29 (23–39)	25 (17–35)
Height (cm)	169 (156–185)	172 (156–188)	170 (154–196)
Top speed (km/h)	6.5 (5–8)	6.6 (4–8)	7.1 (5.5–8)
Pre-op OA severity	3 (2–4)	3 (2–4)	NA
Follow-up (months)	13 (12–15)^‡^	20 (13–30)	NA
Oxford hip score	46 (43–48)	46 (40–48)	NA
EQ-5D	0.88 (0.62–1)	0.92 (0.72–1)	NA
EQ-VAS	85 (50–100)	83 (50–100)	NA

The values are indicated as means (range); ^†^ significant difference between patient groups versus control (*p* < 0.05); ^‡^ significant difference between patient groups (*p* < 0.05)

### Gait analysis

The mean top walking speed was 6.5 and 6.6 km/h for the short and long, respectively. This was 9% slower than the control group who walked at a mean of 7.1 km/h. This difference however failed to reach significance (*p* = 0.09 and 0.11) for long and short groups respectively.

**Fig. 3:**
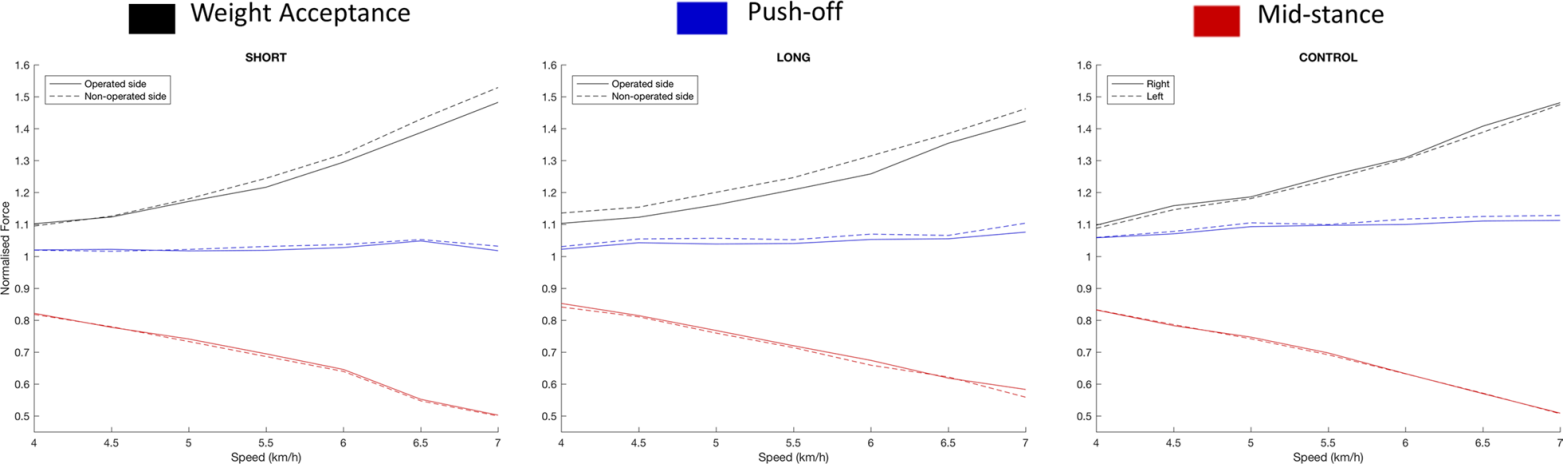
Ground reaction force trends on the flat

**Fig. 4:**
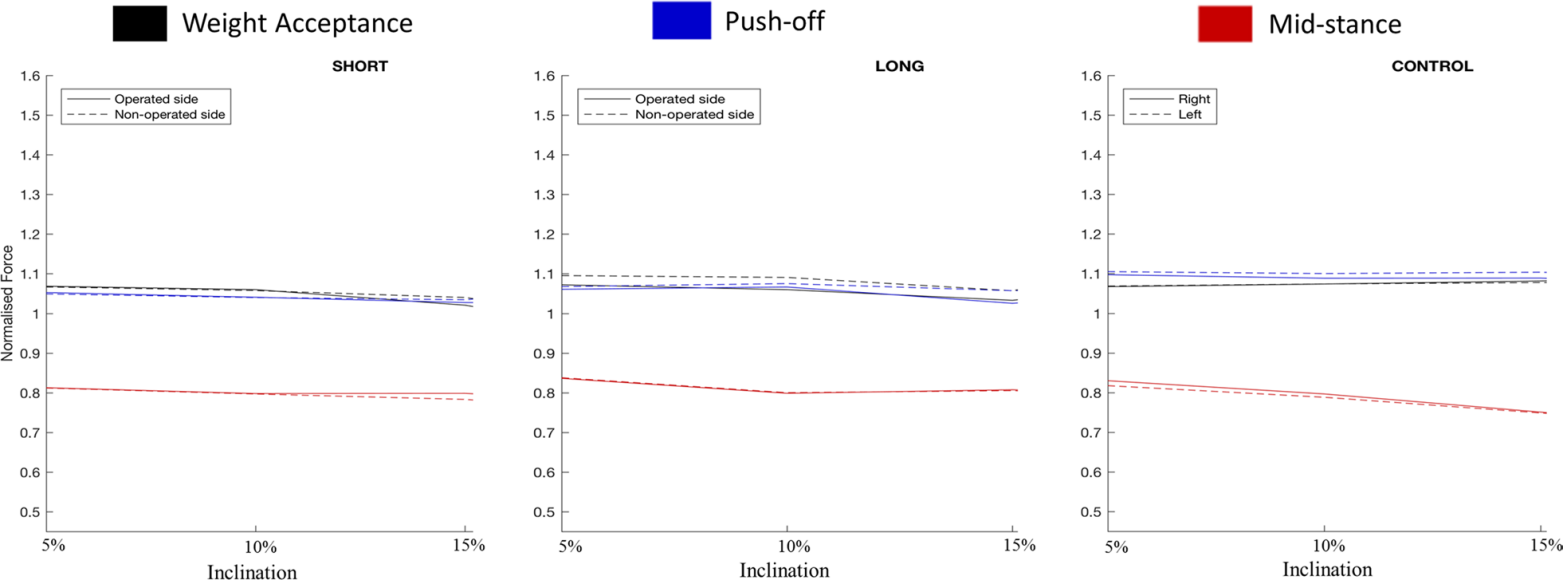
Ground reaction force trends on inclines

Both hip implanted groups largely walked within a normal range of asymmetry for weight acceptance, mid-stance, push-off and step length (Tables 2, 3 and Fig. 3, 4). The only found difference in asymmetry was detected for the short stem device on the flat at 6.5 km/h during weight acceptance (*p* = 0.049) and at 7.0 km/h during mid-stance (*p* = 0.025).

**Table 2:** Flat speed parameters means with mean absolute symmetry indices (SI) in percent %

Variable	Speed		4 km/h	SI	4.5 km/h	SI	5 km/h	SI	5.5 km/h	SI	6 km/h	SI	6.5 km/h	SI	7.0 km/h	SI
	Groups	Limb														
Weight-acceptance (BWN)	Short stem THR	Implanted	1.10 (1.00–1.21)	2.6	1.12 (0.98–1.30)	2.9	1.17 (0.99–1.36)	3.6	1.22 (1.04–1.43)	4.4	1.30 (1.15–1.56)	5.4	1.39 (1.21–1.73)	6.9^†^	1.48 (1.28–1.66)	6.2
		Non-implanted	1.10 (1.02–1.18)		1.13 (1.00–1.26)		1.18 (1.03–1.34)		1.24 (1.08–1.38)		1.32 (1.21–1.46)		1.43 (1.26–1.61)		1.53 (1.35–1.63)	
	Long stem THR	Implanted	1.10 (1.03–1.18)	4.2	1.12 (1.04–1.26)	4.3	1.16 (1.05–1.28)	5.0	1.21 (1.11–1.30)	4.5	1.26 (1.16–1.39)	4.8	1.35 (1.20–1.49)	3.1	1.42 (1.31–1.61)	6.6
		Non-implanted	1.13 (1.04–1.22)		1.15 (1.00–1.29)		1.20 (1.04–1.31)		1.25 (1.08–1.37)		1.31 (1.18–1.50)		1.38 (1.22–1.54)		1.46 (1.34–1.62)	
	Control	Right	1.10 (0.99–1.39)	2.9	1.16 (1.05–1.51)	3.0	1.19 (1.08–1.39)	3.4	1.25 (1.14–1.40)	2.8	1.31 (1.18–1.47)	3.1	1.41 (1.28–1.60)	3.2	1.48 (1.36–1.72)	3.5
		Left	1.09 (0.97–1.40)		1.15 (1.00–1.44)		1.18 (1.05–1.32)		1.24 (1.14–1.38)		1.31 (1.20–1.44)		1.39 (1.28–1.60)		1.47 (1.34–1.62)	
Mid-stance (BWN)	Short stem THR	Implanted	0.82 (0.77–0.88)	2.8	0.78 (0.72–0.84)	3.2	0.74 (0.69–0.79)	3.6	0.69 (0.64–0.76)	3.6	0.65 (0.57–0.77)	5.4	0.55 (0.48–0.64)	8.5	0.50 (0.38–0.62)	11.6^†^
		Non-implanted	0.82 (0.78–0.86)		0.78 (0.74–0.86)		0.73 (0.64–0.82)		0.69 (0.62–0.78)		0.64 (0.52–0.81)		0.54 (0.41–0.60)		0.49 (0.34–0.57)	
	Long stem THR	Implanted	0.85 (0.79–0.91)	3.1	0.81 (0.75–0.90)	3.0	0.77 (0.67–0.86)	4.1	0.71 (0.64–0.84)	4.4	0.67 (0.57–0.80)	4.2	0.62 (0.50–0.70)	7.2	0.58 (0.45–0.69)	9.1
		Non-implanted	0.84 (0.75–0.90)		0.81 (0.74–0.92)		0.76 (0.66–0.86)		0.70 (0.63–0.86)		0.66 (0.55–0.81)		0.62 (0.51–0.78)		0.55 (0.47–0.70)	
	Control	Right	0.83 (0.75–0.93)	2.9	0.78 (0.68–0.87)	3.1	0.75 (0.62–0.85)	3.0	0.72 (0.56–0.82)	3.6	0.63 (0.49–0.77)	4.3	0.57 (0.47–0.73)	5.2	0.51 (0.36–0.66)	5.8
		Left	0.83 (0.74–0.95)		0.79 (0.69–0.88)		0.74 (0.65–0.86)		0.70 (0.57–0.79)		0.63 (0.48–0.75)		0.57 (0.40–0.74)		0.51 (0.37–0.66)	
Push-off (BWN)	Short stem THR	Implanted	1.02 (0.93–1.16)	3.2	1.02 (0.94–1.14)	3.2	1.02 (0.90–1.16)^†^	3.8	1.02 (0.88–1.18)^†^	3.5	1.03 (0.85–1.23)^†^	4.0	1.05 (0.77–1.25)	4.5	1.02 (0.77–1.25)	3.4
		Non-implanted	1.02 (0.93–1.17)		1.02 (0.91–1.18)		1.03 (0.88–1.24)^†^		1.03 (0.86–1.28)^†^		1.04 (0.83–1.36)^†^		1.05 (0.79–1.38)		1.03 (0.78–1.34)	
	Long stem THR	Implanted	1.02 (0.92–1.15)	2.8	1.04 (0.87–1.17)	2.1	1.03 (0.85–1.19)^†^	2.6	1.04 (0.88–1.18)^†^	3.3	1.05 (0.92–1.19)	4.3	1.06 (0.88–1.19)	4.3	1.07 (1.00–1.17)	4.9
		Non-implanted	1.03 (0.88–1.18)		1.05 (0.89–1.19)		1.06 (0.81–1.21)^†^		1.05 (0.81–1.20)^†^		1.07 (0.91–1.26)		1.07 (0.86–1.28)		1.10 (0.98–1.30)	
	Control	Right	1.06 (0.93–1.25)	3.0	1.07 (0.89–1.24)	2.3	1.09 (0.96–1.26)	3.3	1.10 (0.96–1.28)	3.1	1.10 (0.93–1.24)	3.6	1.11 (0.90–1.26)	3.6	1.11 (0.93–1.31)	4.6
		Left	1.06 (0.90–1.20)		1.08 (0.88–1.25)		1.10 (0.96–1.27)		1.10 (0.93–1.27)		1.12 (0.92–1.28)		1.12 (0.94–1.30)		1.13 (0.90–1.36)	
Step length to height (N)	Short stem THR	Implanted	0.60 (0.54–0.69)	3.5	0.65 (0.59–0.69)	3.3	0.70 (0.63–0.76)	2.8	0.75 (0.66–0.83)	2.8	0.78 (0.66–0.87)	2.2	0.82 (0.76–0.88)	1.7	0.86 (0.78–0.90)	2.5
		Non-implanted	0.60 (0.54–0.68)		0.65 (0.57–0.72)		0.70 (0.64–0.76)		0.75 (0.66–0.81)		0.78 (0.70–0.86)		0.84 (0.78–0.88)		0.85 (0.80–0.90)	
	Long stem THR	Implanted	0.60 (0.51–0.69)	2.9	0.66 (0.56–0.74)	2.7	0.70 (0.61–0.80)	3.3	0.75 (0.65–0.85)	3.3	0.79 (0.69–0.86)	2.5	0.83 (0.79–0.90)	3.2	0.87 (0.78–0.90)	2.7
		Non-implanted	0.59 (0.50–0.68)		0.65 (0.56–0.74)		0.70 (0.59–0.83)		0.75 (0.63–0.88)		0.79 (0.63–0.86)		0.82 (0.65–0.89)		0.86 (0.77–0.91)	
	Control	Right	0.63 (0.53–0.74)	2.7	0.68 (0.56–0.83)	2.8	0.73 (0.65–0.85)	2.0	0.77 (0.74–0.94)	2.0	0.81 (0.70–0.97)	2.0	0.84 (0.75–1.02)	2.1	0.87 (0.77–0.99)	2.5
		Left	0.63 (0.54–0.74)		0.67 (0.56–0.78)		0.73 (0.63–0.84)		0.77 (0.69–0.90)		0.81 (0.72–0.96)		0.84 (0.75–1.02)		0.87 (0.74–0.99)	

Values are presented as means (range). SI signifies absolute symmetry index in percent %. BWN signifies body weight normalized force

*N* signifies normalised to height as in step length. ^†^ Significant difference between patient groups versus controls (*p* < 0.05)

**Table 3:** Incline parameters means with mean absolute symmetry indices (SI) in percent %

Variable	Incline		5%	SI	10%	SI	15%	SI
	Groups	Limb						
Weight-acceptance (BWN)	Short stem THR	Implanted	1.07 (0.97–1.07)	2.8	1.06 (0.96–1.12)	4.2	1.02 (0.94–1.22)^†^	4.1
		Non-implanted	1.07 (0.97–1.07)		1.06 (0.95–1.12)		1.04 (0.95–1.18)	
	Long stem THR	Implanted	1.07 (1.02–1.29)	3.3	1.06 (0.96–1.27)	3.7	1.03 (0.95–1.19)^†^	4.0
		Non-implanted	1.10 (0.98–1.29)		1.10 (0.98–1.26)		1.06 (0.92–1.22)	
	Control	Right	1.07 (0.95–1.23)	3.0	1.07 (1.00–1.21)	2.8	1.08 (0.99–1.40)	2.6
		Left	1.07 (0.90–1.17)		1.07 (0.95–1.19)		1.08 (0.99–1.41)	
Mid-stance (BWN)	Short stem THR	Implanted	0.81 (0.74–0.95)	3.5	0.80 (0.70–0.85)	3.0	0.80 (0.75–0.88)	4.2
		Non-implanted	0.81 (0.78–0.93)		0.80 (0.70–0.85)		0.78 (0.72–0.86)	
	Long stem THR	Implanted	0.84 (0.72–0.91)	3.1	0.81 (0.69–0.92)	3.3	0.81 (0.71–0.91)	2.7
		Non-implanted	0.84 (0.74–0.89)		0.80 (0.69–0.88)		0.81 (0.73–0.90)	
	Control	Right	0.83 (0.75–0.89)	3.1	0.80 (0.69–0.89)	3.3	0.75 (0.66–0.93)	4.3
		Left	0.82 (0.74–0.91)		0.79 (0.66–0.87)		0.75 (0.60–0.95)	
Push-off (BWN)	Short stem THR	Implanted	1.05 (0.99–1.09)	3.4	1.04 (0.97–1.14)	2.5	1.03 (0.94–1.16)	3.0
		Non-implanted	1.05 (0.97–1.12)		1.04 (0.97–1.20)		1.03 (0.95–1.17)	
	Long stem THR	Implanted	1.06 (0.96–0.94)	3.0	1.07 (0.98–1.14)	2.7	1.03 (0.92–1.15)	4.7
		Non-implanted	1.07 (0.94–0.94)		1.08 (0.96–1.20)		1.06 (0.96–1.17)	
	Control	Right	1.10 (0.90–1.13)	2.8	1.09 (1.01–1.25)	2.5	1.09 (0.90–1.24)	3.1
		Left	1.11 (0.96–1.12)		1.10 (0.99–1.27)		1.10 (0.91–1.20)	
Step length to height (N)	Short stem THR	Implanted	0.63 (0.55–0.72)	3.6	0.62 (0.55–0.71)	3.5	0.61 (0.53–0.69)	4.8
		Non-implanted	0.63 (0.55–0.71)		0.63 (0.56–0.70)		0.62 (0.58–0.68)	
	Long stem THR	Implanted	0.62 (0.53–0.70)	2.6	0.63 (0.54–0.70)	4.8	0.62 (0.55–0.69)	3.5
		Non-implanted	0.62 (0.54–.070)		0.61 (0.52–0.69)		0.61 (0.52–0.69)	
	Control	Right	0.64 (0.57–0.73)	3.2	0.64 (0.55–0.73)	2.9	0.63 (0.51–0.74)	3.6
		Left	0.64 (0.51–0.73)		0.64 (0.54–0.73)		0.63 (0.57–0.74)	

Values are presented as means (range). SI signifies absolute symmetry index in percent %. BWN signifies body weight normalized force

N signifies normalised to height as in step length. ^†^ Significant difference between patient groups versus controls (*p* < 0.05)

Examining the operated-hip limb variables in isolation, all prostheses enabled patients to walk within a normal range for weight acceptance, midstance and step length that was indistinguishable from normal controls at all speeds and gradients (Table 2, 3). Despite not being statistically positive, by visualizing the plots through the speeds (Fig. 3), it was clear that hip arthroplasty patients irrespective of design preferred to load the contralateral normal hip and spare the implanted side marginally during weight acceptance. Nevertheless push-off was the variable by which differences were detected (Table 2). Both the shorter and the longer stemmed groups walked with lower push-off forces than the controls on the flat. Statistical differences were found on the flat at 5 km, 5.5 km/h for the long stemmed device (*p* = 0.045, *p* = 0.049 respectively), and at 5, 5.5 and 6 km/h for the short stemmed device (*p* = 0.003, *p* = 0.007, *p* = 0.022 respectively). The weaker push-off did continue to affect uphill walking, particularly on increasing inclines, but this did not reach statistical significance. The only significant difference during uphill was found during 15 degree inclined walking where both hip replacements walked with less weight acceptance than controls (*p* = 0.03 and *p* = 0.045), short and long respectively. After data screening, both hip replacements consistently had less normalised step length, however this did not reach significance for both flat and uphill walking.

## Discussion

This small retrospective gait study set out to determine the loading patterns of differing femoral stem lengths at a variety of speeds and inclines to detect a difference. We sought to establish the effect of a similarly designed shorter stem in comparison to a traditional length ODEP 10A* rated conventional stem. The strengths of this study include the strict inclusion–exclusion criteria and having a comparable healthy cohort. In spite of lack of randomisation this study only identified patients primarily with unilateral simple coxarthrosis with subsequent replacement and no other disease to affect gait. The implant groups were uniform, with a single device design, a single approach and rehabilitation regime and a single surgeon. The patient groups were similar as possible with regards to demographics, with identical postoperative Oxford Hip Scores, pre-operative radiographic diagnosis and severity.

The fundamental limitation is the significantly shorter mean follow-up (13 vs 21 months) for the short stem when compared to the long stem THR. However a prized double blinded randomised study comparing hip resurfacing versus large head THR found operated patients to reach near control gait parameters as early as 3 months post-operatively with the 12 month assessment demonstrating indistinguishable gait compared to healthy controls [26]. Our study also did lack pre-operative analysis, which did not permit differences in improvement to be measured, which could offer further important knowledge. Nonetheless the intention of this single snapshot study, with a non-inferiority outcome, was primarily to assess the loading behaviour of a short stem THA when compared to the contralateral healthy side.

The most obvious finding of this study was that both THA groups with near perfect PROMs could not achieve a top walking speed of healthy controls. This reconfirms the ceiling effects of PROMs and highlights the importance for the need of other metrics to detect differences in performance following hip arthroplasty.

Walking speed is an identifiable and reproducible measure of clinical relevance [27] but selection bias is hard to avoid [28]. A more robust metric of performance was therefore sought: the symmetry of gait. This allows us to observe the impact of implant type on the force delivered by that limb at any speed or incline, compared to the un-diseased contralateral side, and to a similar aged cohort of asymptomatic controls as a benchmark. An absolute value—the symmetry index—was used to prevent any bias disguised by the averaging effect of different directions of the symmetry mean. This was prompted by the earlier report that different types of arthroplasty deliver different amounts of force [16].

The most positive finding of this study was that both hip implants irrespective of stem length enabled the limb to load to a normal range of asymmetry with regards to weight acceptance, mid-stance, and push-off at differing speeds and gradients. This is in contrast to the published impact of any form of knee arthroplasty on gait, where substantial departures from the normal gait are found with all implant types tested [29]. The data we present confirms that all forms of hip arthroplasty enable a near physiological heel strike, and loading during early stance phase. This suggests that these patients were comfortable loading in the flexion phase of stance of these hip devices throughout the range of walking speeds and incline. The only differences detected between the groups were noted at late stance phase, particularly at push-off. Significantly reduced push-off was seen in both stemmed groups when compared to the controls for overall push-off force. Push-off occurs on the treadmill when the hip is in the terminal phase of extension during walking. This observation could mean either that the presence of a stem does not allow appropriate loading during push-off, or perhaps that they were simply weaker. The latter may well be the reason as they were significantly slower at top walking speed when compared to the controls that had higher push-off forces. The fact that the shorter stems were associated with marginally worse push-off is interesting. However being substantially older could account for this. Nevertheless all groups’ push-off forces were similar in terms of symmetry and reflect the intended outcome of a stable hip device when compared to the contra-lateral normal side.

The data we present cannot determine the exact cause of the weakness with push-off found with stemmed hip arthroplasties. Reduction in muscle mass and strength in an ageing and arthritic population are associated with reduced push-off [29], but inherent differences in terms of the load transfer between implant stem and femoral shaft cannot not be excluded as a reason [30].

The analysis we present did not focus solely on top walking speed but rather the entire range of gait which gives the reader the general trend of an important and everyday activity. Until now, this has not been reported for a short stem THR moreover differing stem length designs. Of significant interest, we have been able to describe the impact on gait of a new shorter femoral stemmed design at 1 year following surgery. Despite the shorter stemmed group being 6 years or 10% older (69 vs 63 *p* < 0.05) the shorter stems were still able to perform as well as the longer stemmed device which has a 10A* rating. Thus the short stems appear to be safe, so the retained compliance of the femur should also be an asset in the longer term, by reducing the risk of periprosthetic fracture [31].

In conclusion, this retrospective comparative study has demonstrated the impact of different femoral stem lengths in THA on gait at differing speeds and inclines. It has revealed the near symmetrical function of patients with a new short femoral stem THA and encouragingly demonstrates no disadvantage when compared to a well-established long femoral stem device. It confirms that the Evolution short stemmed THA is safe in human hips in the short term and may come as an attraction to long stemmed user who is interested in maintaining the flexibility of the proximal femur in the long term.
